# Could impulse oscillometry be an easy and practical test for differential diagnosis in healthy adults and patients with asthma and COPD?

**DOI:** 10.1097/MD.0000000000048237

**Published:** 2026-04-03

**Authors:** Buket Caliskaner Ozturk, Ilgim Vardaloglu, Enes Furkan Aykac, Nihal Ensen, Gunay Can, Sermin Borekci, Bilun Gemicioglu

**Affiliations:** a Department of Pulmonary Diseases, Cerrahpasa Faculty of Medicine, Istanbul University-Cerrahpasa, Istanbul, Turkey; b Department of Public Health, Cerrahpasa Faculty of Medicine, Istanbul University-Cerrahpasa, Istanbul, Turkey.

**Keywords:** asthma, COPD, oscillometry

## Abstract

Impulse oscillometry (IOS) is a type of oscillation technique that can detect pathological changes in small airways early. While spirometry is still normal in asthma and in patients who will have chronic obstructive pulmonary disease (COPD) in future, IOS can detect increased airway resistance with increased sensitivity in the early stages. The aim of this study is to evaluate IOS in healthy adults and patients with asthma and COPD. In this prospective and observational study; healthy adults without any airway disease and patients with asthma and COPD admitted to the pulmonology outpatient clinic were included. The IOS and spirometry tests were performed simultaneously in all 3 groups, and the test results and demographic data were recorded. Higher AX (*kPa/L*), Fres (*1/s*), lower X5 Hz (kPa/[*L/s*]), FEV1(*L*), FEV1/FVC%, MEF 25–75 (*L*), FEV3 (*L*), FEV3/FEV6%, FEV3/FVC6% values were found in COPD and asthma patients compared to healthy adults. The resistance difference between 5 and 20 Hz was unaffected in asthma patients whereas it was higher in COPD patients than in healthy adults. R5 Hz (kPa/[*L/s*]) was significantly higher in COPD patients than in healthy and asthma patients (*P* = .01). Our study results suggest that IOS shows significant variations among healthy adults, asthma patients, and those with COPD.

## 1. Introduction

Impulse oscillometry (IOS) is a type of oscillation technique and is a pulmonary function test that evaluates lung mechanics. It gives information about resistance (R) and extensibility (reactance-X) due to oscillating pressureflow relationships using sound waves during normal tidal breathing. Resistance and reactance are measured and named between specific frequencies (5–20 Hz).

Impulse oscillometry can be performed with minimal cooperation, regardless of patient effort.^[1]^ As it is easier to apply than spirometry, the results contain less error.^[2]^ Although it is independent of patient effort, IOS variables are prone to errors from not proper seal at mouth and glottal closure.^[3]^ Impulse oscillometry can distinguish small from large airway obstruction and is more sensitive than spirometry for peripheral airway disease.^[4]^ Therefore, it can detect pathological changes early by measuring the increased resistance in the small airways, when there are no symptoms and spirometry is normal in the initial period of airway diseases.^[5,6]^ Moreover, it is superior to spirometry in detecting the absence of airway pathology.^[7]^

Most asthma patients have preserved lung functions on spirometric measurement. Therefore, in addition to medical history and physical examination, several methods such as reversibility tests, bronchial provocation tests, sputum cytology, and fractionated extra nitric oxide may be required to diagnose patients. Access and applicability to these additional tests are not easy. At this point, IOS stands out with its easy applicability. It can help to identify asthma patients by detecting airway resistance and reactance. IOS has also been used during bronchoprovocation tests, and some patients have seen positive results on IOS without significant changes in FEV1.^[8]^

Previous studies have supported that oscillometry is a method to be used safely instead of spirometry in patients with chronic obstructive pulmonary disease (COPD).^[9,10]^ It has also been shown to contribute clinically to the detection of emphysema in COPD patients.^[11]^ While spirometry is normal in asymptomatic smokers, small airway dysfunction has been demonstrated with the help of IOS.^[12]^

In order to determine the reference value in adults, 882 studies conducted in the last 40 years were examined and it was concluded that the existing literature providing reference values and prediction equations for respiratory impedance measurements is limited.^[13]^ Currently, we still do not have the results IOS reference values can accept. In the present study, we, therefore, aimed to evaluate IOS in healthy adults and patients with asthma and COPD.

## 2. Materials and methods

### 2.1. Study design

This study is a prospective, observational study. The study was approved by Ethics Committee of Istanbul University-Cerrahpasa, Cerrahpasa Faculty of Medicine (E-83045809-604.01.02-76091, Date: April 20, 2021) and conducted in accordance with the principles of the Declaration of Helsinki. Prior to study, a written informed consent was obtained from each participant.

### 2.2. Setting

Between June 1, 2022 and December 31, 2022, 55 healthy volunteers who met the criteria and 41 stable asthma and 57 COPD patients followed in the university chest diseases outpatient clinic were included in the study.

### 2.3. Participants

#### 2.3.1. Inclusion criteria

For the healthy volunteer group, being older than 30 years, younger than 65 years old, and not having any lung disease.

For the asthma group, being older than 30 years, younger than 65 years old, diagnosed with asthma according to The Global Initiative for Asthma (GINA) Strategy Report 2021 criteria, never used short acting bronchodilators for at least 6 hours before the test, never used long-acting bronchodilators for least 24 hours.^[14]^

For the COPD group, being older than 30 years, younger than 65 years old, having a diagnosis of COPD according to The Global Initiative for Chronic Obstructive Lung Disease (GOLD) report 2021 criteria, not using short acting bronchodilators for at least 6 hours before the test, long-acting beta-agonists for at least 24 hours, and ultra long-acting beta-agonists and anticholinergics for at least 36–48 hours.^[15]^

#### 2.3.2. Exclusion criteria

*For all groups;* not giving consent to the voluntary consent form.

*For COPD and asthma group;* Having a history of systemic steroid use in the last 1 month, being in an attack or having an attack in the last 1 month.

### 2.4. Variables

Patients’ age, sex, body mass index (BMI), smoking history, IOS and pulmonary function test parameters were recorded.

### 2.5. Data sources/measurement

#### 2.5.1. IOS and spirometry measurement

All respiratory function tests were performed on each patient on the same day. During the tests, each patient first underwent IOS, then underwent spirometry.

Oscillometry measurements were performed with the CareFusion IOS Vyntus® 42201238 device (CareFusion Corp., Würzburg, Germany) according to the standard recommended by the European Respiratory Society (ERS).^[16]^ Participants were asked to do tidal breathing for 30 to 40 seconds through a mouthpiece connected to a loudspeaker that produced pressure oscillations of multiple frequencies. Participants were asked to sit upright on a chair, put clips on their noses, and firmly support their cheeks with both hands. At least 3 attempts were made according to the recommended ERS standard. The mean values from the 3 IOS measurements were recorded. R5, R20, X5, AX, and Fres parameters were evaluated. In our study, European data were taken as reference like in other studies for the R5 to R20 value.^[17]^

Spirometry was measured with the CareFusion IOS Vyntus® 42201238 device, according to the spirometry standardization rules jointly determined by the ERS and American Thoracic Society.^[18]^ Forced expiratory volume in 1st second (FEV1) (L), forced vital capacity (FVC) (L), FEV1/FVC%, maximal mid expiratory flow (MEF) 25 to 75(L), forced expiratory volume in 3rd second (FEV3) (L), forced expiratory volume in 6th second (FEV6) (L), FEV3/FEV6%, FEV3/FVC%, (FVC-FEV3)/FVC%, (FEV6-FEV3)/FVC% were checked.

### 2.6. Biass

While creating the healthy adult group, the health systems of all patients were scanned retrospectively and respiratory examinations were performed by a pulmonologist.

### 2.7. Study size

A total of 153 individuals, 55 patients in the healthy volunteer group, 41 patients in the asthma group, and 57 patients in the COPD group, were included in the study between the specified dates. We included all patients applied between June 1, 2022 and December 31, 2022 who met our inclusion criteria. Our post hoc power analysis revealed that Fres, AX, and R5 values were above 0.90.

### 2.8. Statistical methods

Statistical analysis was performed using the SPSS version 21.0 software (IBM Corp., Armonk). Descriptive data were expressed in mean ± standard deviation or number and frequency, where applicable. Analysis of variance (ANOVA) was used for data with normal distribution to compare quantitative data between groups. Comparisons of pulmonary function test values between groups were evaluated with the Kruskal–Wallis–Dunn tests and covariance analysis (age, sex, BMI, and smoking). The correlations between FEV3 and other FEV3-related spirometry parameters and R5 to R20 Hz were analyzed according to age, BMI, and cigarette smoking. A *P*-value of <.05 was considered statistically significant.

## 3. Results

### 3.1. Participants

Among the patients who applied to the pulmonary diseases outpatient clinics during the study period for the asthma and COPD group, 255 asthma and 215 COPD patients were evaluated for the study. Among them, 44 were found to meet the criteria for the asthma group, and 3 were excluded, as they did not give consent for participation. For the COPD group, 65 met the criteria, of which 8 were excluded, as they did not give consent to participate. Forty one patients for the remaining asthma group and 57 patients for the COPD group were included in the study. For the healthy volunteer group, 55 individuals who met the criteria and gave consent were included in the study

### 3.2. Descriptive data

Of the 153 participants, 55 were healthy adults, 41 had asthma, and 57 had COPD. The mean age of the groups was 45.24 ± 13.02, 45.51 ± 16.17, and 61.21 ± 9.04, respectively. Sex ratios and smoking histories are presented in Table 1.

**Table 1:** Demographic characteristics.

	Healthy (n = 55)	Asthma (n = 41)	COPD (n = 57)	*P*
	Mean ± SD, N (%)	Mean ± SD, N (%)	Mean ± SD, N (%)	
Age (year)	45.24 ± 13.02	45.51 ± 16.17	**61.21 ± 9.04**	**.001**
Sex				
Male	29 (52.7%)	16 (39%)	21 (77.7%)	.61
Female	26 (47.3%)	25 (61%)	6 (33.3%)	–
BMI (kg/m^2)^	26.97 ± 5.2	27.83 ± 6	27.35 ± 5.85	.76
Smoking (package year)	10.5 ± 5.21	8.32 ± 8.74	29.85 ± 15.25	**.001**

Values that are statistically significant are presented in bold.

BMI = body mass index, COPD = chronic obstructive pulmonary disease, SD = standard deviation.

### 3.3. Outcomes and main data

In the healthy adult group, significant differences were found in values AX (kPa/L), X5 Hz (kPa/[L/s]), Fres (1/s), FEV1(L), FEV1/FVC %, MEF 25–75 (L), FEV3 (L), FEV3/FEV6%, according to both asthma and COPD patients. In healthy adults, R5 Hz (kPa/[L/s]) was significantly lower than COPD patients and AX (kPa/L) was significantly lower than asthma and COPD patients (*P* <.001), X5 Hz (kPa/[L/s]), FEV1(L), FEV1/FVC%, MEF 25–75 (L), FEV3 (L), FEV3/FEV6%, FEV3/FVC% values were found to be significantly higher and Fres (1/s) was found to be significantly lower than asthma and COPD patients (*P* <.05) (Table 2).

**Table 2:** Comparison of healthy, COPD and asthma groups.

	Healthy			Asthma			COPD			*P*
	Median	IQR25	IQR75	Median	IQR25	IQR75	Median	IQR25	IQR75	
R5 Hz (kPa/[L/s])	0.4	0.3	0.5	0.4	0.4	0.7	**0.5**	**0.4**	**0.7**	**.01**
R20 Hz (kPa/[L/s])	0.3	0.3	0.4	0.4	0.3	0.4	0.3	0.3	0.4	.38
R5–R20 Hz (kPa/[L/s])	0.04	0.02	0.09	0.08	0.05	0.2	**0.2**	**0.03**	**0.4**	**.007**
(R5–R20)/R5%	13	8	23	21	11	38	39	09	58	**.03**
AX (kPa/L)	0.33	0.18	0.75	**0.89**	**0.26**	**2.67**	**1.98**	**0.39**	**4.30**	**0 <001**
X5 Hz (kPa/[L/s])	**−0.08**	**−0.11**	**−0.05**	−0.12	−0.19	−0.07	−0.11	−0.28	−0.08	**0 <001**
Fres (1/s)	14	10.9	19	**19**	**12.9**	**30.6**	**24.8**	**19.3**	**30.9**	**0 <001**
FEV1 (L)	**3.04**	**2.46**	**3.57**	2.34	1.65	3.46	1.91	1.25	3.13	**0 <001**
FEV1%	**102**	**92**	**111**	**96**	**81.5**	**106.5**	59	44	86	**0 <001**
FVC (L)	3.62	3.17	4.38	3.25	2.54	4.27	3.59	2.50	4.41	.11
FVC%	**103**	**93**	**119**	**104**	**96.5**	**117.5**	85	69	109	**.009**
FEV1/FVC%	**84**	**77.5**	**88.7**	**73**	**61.7**	**81.5**	57	41.6	68	**.000**
MEF25–75 (L)	**3**	**2**	**3.8**	1.46	0.61	2.74	0.6	0.25	13.5	**0 <001**
MEF25%–75%	**84.5**	**69.2**	**95.7**	49	23	75	21.5	11.2	38.2	**0 <001**
FEV3 (L)	**3.42**	**2.88**	**4.07**	2.74	2.05	3.79	2.78	1.83	3.93	**.003**
FEV6 (L)	3.54	3	4.29	2.83	2.3	3.79	3.08	2.11	4.23	.054
FEV3/FEV6%	**95**	**94**	**97**	**93**	**89**	**94**	87	83	90	**0 <001**
FEV3/FVC%	**95**	**92**	**97**	**90**	**85**	**92**	78	69	89	**0 <001**
(FVC–FEV3)/FVC%	5	3	8	**10**	**8**	**15**	**22**	**11**	**31**	**0 <001**
(FEV6–FEV3)/FVC%	4	2	6	6	5	10	**11**	**09**	**15**	**0 <001**

Values that are statistically significant are presented in bold.

AX = area under the reactance curve, FEV 1 = forced expiratory volume in the first second, FEV3 = forced expiratory volume in 3 seconds, FEV6 = forced expiratory volume in 6 seconds, Fres = resonant frequency, FVC = forced vital capacity, MEF 25%–75% = maximal mid expiratory flow, R5 = resistance at 5 Hz, R20 = resistance at 20 Hz, R5–R20 = difference in resistance between R5 and R20, X5 Hz = reactance at 5 Hz.

In asthma patients, R5 to R20 Hz was similar to healthy adults, while it was significantly higher in COPD patients than in healthy adults (*P* = .07). R5 Hz was significantly lower in asthma patients than in COPD patients (*P* = .01).

**Figure 1.**
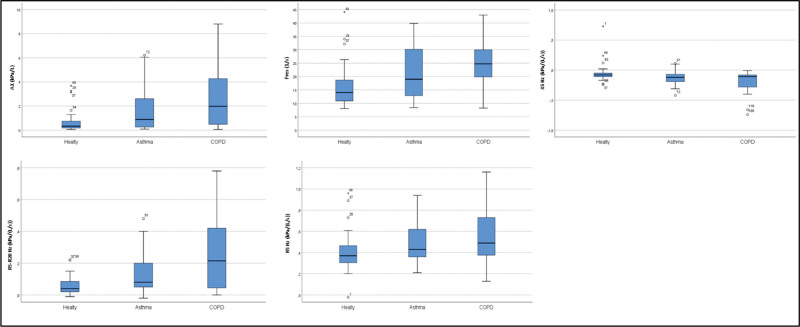
Comparison of healthy, COPD and asthma groups. AX = area under the reactance curve, COPD = chronic obstructive pulmonary disease, Fres = resonant frequency, R5 = resistance at 5 Hz, R5–R20 = difference in resistance between R5 and R20, X5 Hz = reactance at 5 Hz.

There was no statistically significant difference between the groups in R20 Hz (kPa/[L/s]), FVC (L), and FEV6 (L) values (*P* >.05) (Table 2, Fig. 1).

**Figure 2.**
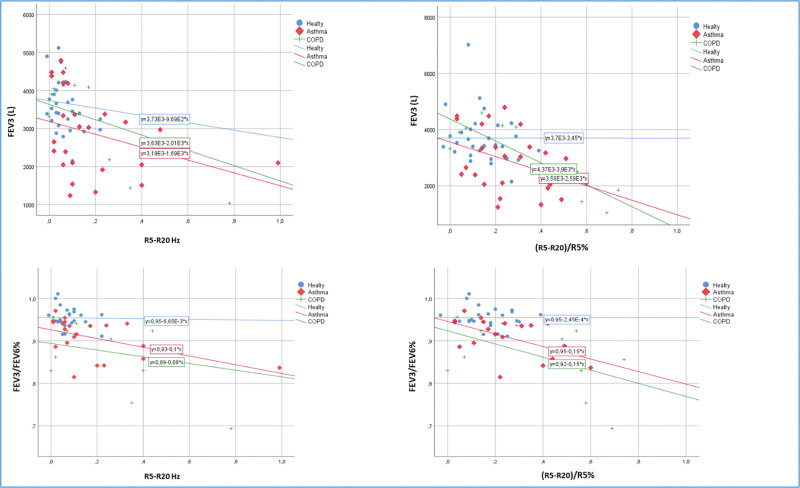
Corelation analysis between FEV3, FEV3/FEV6 and R5–R20, R5%–R20%/R5%. COPD = chronic obstructive pulmonary disease, FEV3 = forced expiratory volume in 3 seconds; FEV6 = forced expiratory volume in 6 secondsR5 = resistance at 5 Hz, R5–R20 = difference in resistance between R5 and R20.

There was no correlation between R5 to R20 Hz, R5% to R20%, and FEV3, other FEV3-related parameters in healthy adults (*P* >.05). In asthma patients, a moderately significant negative correlation between R5 to R20 Hz, R5% to R20% and FEV3/FEV6%, FEV3/FVC% and a moderately significant positive correlation between (FVC–FEV3)/FVC% and (FEV6–FEV3)/FVC% were detected (*P* <.05, *r* >.50) (Table 3). In COPD patients, there was a strong and statistically significant negative correlation between R5 to R20 Hz, R5% to R20% and FEV3, FEV3/FEV6%, FEV3/FVC%, while (FVC–FEV3)/FVC% and (FEV6–FEV3)/FVC% A strong and statistically significant positive correlation was found between R5 to R20 Hz and R5% to R20% (*P* <.01, *r* >.75) (Table 3, Fig. 2).

**Table 3:** Correlation analysis between FEV3 and R5 to R20.

		FEV3 (L)		FEV3/FEV6%		FEV3/FVC%		(FVC-FEV3)/FVC%		(FEV6-FEV3)/FVC%	
		*r*	*P*	*r*	*P*	*r*	*P*	*r*	*P*	*r*	*P*
Healty	R5–R20 Hz	−0.11	.65	0.02	.94	0.08	.74	−0.08	.74	−0.02	.95
	R5%–R20%	0.18	.45	0.01	.97	0.10	.68	−0.10	.68	−0.01	.98
Asthma	R5–R20 Hz	−0.32	.20	**−0.54**	**.02**	**−0.57**	**.01**	**0.57**	**.01**	**0.52**	**.03**
	R5%–R20%	−0.32	.20	**−0.56**	**.02**	**−0.54**	**.02**	**0.54**	**.02**	**0.56**	**.02**
COPD	R5–R20 Hz	**−0.87**	**.001**	**−0.83**	**.01**	**−0.88**	**.00**	**0.88**	**.001**	**0.79**	**.01**
	R5%–R20%	**−0.94**	**.001**	**−0.87**	**.001**	**−0.91**	**.00**	**0.91**	**.001**	**0.80**	**.01**

Values that are statistically significant are presented in bold.

COPD = chronic obstructive pulmonary disease, FEV3 = forced expiratory volume in 3 s, FEV6 = forced expiratory volume in 6 s, FVC = forced vital capacity, R5–R20 = difference in resistance between R5 and R20.

## 4. Discussion

After IOS and spirometry tests, higher AX (kPa/L) and Fres (1/s), lower X5 Hz (kPa/[L/s]), FEV1(L), FEV1/FVC%, MEF 25–75 (L), FEV3 (L), FEV3/FEV6%, FEV3/FVC6% values were found in COPD and asthma patients compared to healthy adults. R5 Hz (kPa/[L/s]) was significantly higher in COPD than patients with asthma. The difference in resistance between 5 and 20 Hz (R5–20 Hz) was insignificant in asthma patients and higher in COPD patients than in healthy adults.

In their study, Wei X et al showed that IOS was an alternative method to spirometry for COPD patients; IOS parameters for COPD patients showed a good correlation with traditional pulmonary function parameters, and both resistance and reactance parameters were affected. Similarly, in this study, resistance and recurrence values showed significant differences from healthy adults.^[9]^

In the study of Li Y et al that used the combination of IOS and bronchial dilation test for the differential diagnosis of asthma and COPD patients, X5, X20, X25, X35, and Fres correlated better with COPD and found that particularly X5, Fres, and X25 were significantly associated with COPD.^[19]^ In our study, X25 was not measured, but X5 Hz values in COPD patients were significantly lower than in healthy individuals and asthma patients. Accordingly, we can speculate that both resistance and recurrence are significantly affected in COPD and are detected in IOS parameters. There are other studies supporting this result^[9]^

Su ZQ et al evaluated the parenchymal findings in Thorax computed tomography with IOS.^[20]^ It supported that the IOS parameters Fres and R5 to R20 Hz are sensitive parameters indicating small airway pathology in COPD. Similarly, in this study, we found that Fres decreased and R5 to R20 increased in COPD patients. In addition, AX was found to be increased in COPD patients.

Furthermore, IOS is used more common in asthma patients than in other patient groups. David W et al showed that R5 and Fres values increased in asthma patients, while X5 decreased.^[21]^ Similarly, in this study, Fres values were higher in asthma patients compared to healthy adults, but the R5 Hz value was not significantly different. The X5 Hz value was found to be lower. In addition, there was no significant difference between Fres and X5 Hz values in COPD and asthma patients. R5 Hz value was higher in COPD patients than in asthma patients.

Several studies have shown a higher increase in R5 than R20 in IOS values in COPD patients; therefore, R5–R20 increases.^[22–25]^ Similarly, both R5 to R20 Hz values were significantly higher than healthy adults in our study. In addition, R5 to R20/R5% was also significantly higher in COPD patients than in healthy ones in this study. Among asthma and COPD patients, no statistically significant difference was found.

Strengths of this study include its comparative examination of IOS and spirometry parameters in asthma, COPD, and healthy controls, which helped identify differences in respiratory functions. In addition, analysis of small airway parameters and correlations between IOS and spirometric values supported the notion that IOS is a reliable and complementary method for assessing respiratory function.

### 4.1. Limitations

Nonetheless, there are some limitations to this study. The main limitation is that it presents general results without classification of asthma and COPD patients according to their treatment. However, we believe that different results would probably be observed on the basis that patients are grouped according to their treatment. Another issue is that asthma and COPD patients were evaluated without grouping according to their severity, due to the limited number of patients in the groups, we could not make such a subgrouping.

## 5. Conclusion

Our study results suggest that IOS shows significant variations among healthy adults, asthma patients, and those with COPD. Considering its ease of use, IOS can be regarded as a function test in the diagnosis and differential diagnosis of airway diseases in cases spirometry cannot be performed.

## Author contributions

**Conceptualization:** Buket Caliskaner Ozturk, Sermin Borekci.

**Data curation:** Buket Caliskaner Ozturk.

**Formal analysis:** Gunay Can, Sermin Borekci.

**Investigation:** Ilgim Vardaloglu, Enes Furkan Aykac, Nihal Ensen.

**Methodology:** Buket Caliskaner Ozturk, Gunay Can, Bilun Gemicioglu.

**Project administration:** Buket Caliskaner Ozturk, Nihal Ensen, Bilun Gemicioglu.

**Resources:** Ilgim Vardaloglu, Enes Furkan Aykac, Nihal Ensen.

**Software:** Ilgim Vardaloglu, Enes Furkan Aykac.

**Supervision:** Buket Caliskaner Ozturk, Nihal Ensen, Bilun Gemicioglu.

**Validation:** Buket Caliskaner Ozturk, Nihal Ensen, Gunay Can, Bilun Gemicioglu.

**Visualization:** Buket Caliskaner Ozturk, Gunay Can.

**Writing – original draft:** Buket Caliskaner Ozturk.

**Writing – review & editing:** Buket Caliskaner Ozturk, Sermin Borekci, Bilun Gemicioglu.

## References

[1] Porojan-Suppini N Fira-Mladinescu O Marc M Tudorache E Oancea C. Lung function assessment by impulse oscillometry in adults. Ther Clin Risk Manag. 2020;16:1139–50. Published 2020 Nov 26 33273817 10.2147/TCRM.S275920 PMC7705955

[2] Johnston R PFT blog Observations, opinions and ideas about pulmonary function testing, Oscillometry. 2020. Online website https://www.pftforum.com/blog/?s=oscillometry&submit=Search. Accessed 03 November 2020.

[3] Bikov A Pride NB Goldman MD. Glottal aperture and buccal airflow leaks critically affect forced oscillometry measurements. Chest. 2015;148:731–8. 25742459 10.1378/chest.14-2644

[4] Desiraju K Agrawal A. Impulse oscillometry: the state-of-art for lung function testing. Lung India. 2016;33:410–6. 27578934 10.4103/0970-2113.184875 PMC4948229

[5] Smith HJ Reinhold P Goldman MD. Forced oscillation technique and impulse oscillometry. Eur Respir Monogr. 2005;31:72.

[6] Gallucci M Carbonara P Pacilli AMG di Palmo E Ricci G Nava S. Use of symptoms scores, spirometry, and other pulmonary function testing for asthma monitoring. Front Pediatr. 2019;7:54. 30891435 10.3389/fped.2019.00054 PMC6413670

[7] Al-Mutairi SS Sharma PN Al-Alawi A Al-Deen JS. Impulse oscillometry: an alternative modality to the conventional pulmonary function test to categorise obstructive pulmonary disorders. Clin Exp Med. 2007;7:56–64. 17609877 10.1007/s10238-007-0126-y

[8] Price OJ Ansley L Bikov A Hull JH. The role of impulse oscillometry in detecting airway dysfunction in athletes. J Asthma. 2016;53:62–8. 26291140 10.3109/02770903.2015.1063647

[9] Wei X Shi Z Cui Y. Impulse oscillometry system as an alternative diagnostic method for chronic obstructive pulmonary disease. Medicine (Baltim). 2017;96:e8543. 10.1097/MD.0000000000008543 PMC5704804 29145259

[10] Crim C Celli B Edwards LD; ECLIPSE investigators. Respiratory system impedance with impulse oscillometry in healthy and COPD subjects: ECLIPSE baseline results. Respir Med. 2011;105:1069–78. 21481577 10.1016/j.rmed.2011.01.010

[11] Klitgaard A Løkke A Hilberg O. Impulse oscillometry as a diagnostic test for pulmonary emphysema in a clinical setting. J Clin Med. 2023;12:1547. Published 2023 Feb 15 36836082 10.3390/jcm12041547 PMC9967696

[12] Pisi R Aiello M Frizzelli A. Detection of small airway dysfunction in asymptomatic smokers with preserved spirometry: the value of the impulse oscillometry system. Int J Chron Obstruct Pulmon Dis. 2021;16:2585–90. Published 2021 Sep 14 34548789 10.2147/COPD.S319972 PMC8449545

[13] Kalchiem-Dekel O Hines SE. Forty years of reference values for respiratory system impedance in adults: 1977-2017. Respir Med. 2018;136:37–47. 29501245 10.1016/j.rmed.2018.01.015

[14] Global Strategy for asthma management and prevention. Global Initiative for Asthma. Updated 2021. Available from: https://ginasthma.org/wp-content/uploads/2021/05/GINA-Main-Report-2021-V2-WMS.pdf. Accessed January 15, 2022.

[15] Global Strategy for the diagnosis, management, and prevention of chronic obstructive pulmonary disease; 2022. Available from: https://goldcopd.org/wp-content/uploads/2021/11/GOLD-REPORT-2022-v1.0-12Nov2021_WMV.pdf. Accessed April 12, 2022.

[16] King GG Bates J Berger KI. Technical standards for respiratory oscillometry. Eur Respir J. 2020;55:1900753. Published 2020 Feb 27 31772002 10.1183/13993003.00753-2019

[17] Oostveen E MacLeod D Lorino H; ERS Task Force on Respiratory Impedance Measurements. The forced oscillation technique in clinical practice: methodology, recommendations and future developments. Eur Respir J. 2003;22:1026–41. 14680096 10.1183/09031936.03.00089403

[18] Graham BL Steenbruggen I Miller MR. Standardization of spirometry 2019 update. an official american thoracic society and European respiratory society technical statement. Am J Respir Crit Care Med. 2019;200:e70–88. 31613151 10.1164/rccm.201908-1590ST PMC6794117

[19] Li Y Chen Y Wang P. Application of impulse oscillometry and bronchial dilation test for analysis in patients with asthma and chronic obstructive pulmonary disease. Int J Clin Exp Med. 2015;8:1271–5. Published 2015 Jan 15 25785124 PMC4358579

[20] Su ZQ Guan WJ Li SY. Significances of spirometry and impulse oscillometry for detecting small airway disorders assessed with endobronchial optical coherence tomography in COPD. Int J Chron Obstruct Pulmon Dis. 2018;13:3031–44. Published 2018 Oct 1 30319251 10.2147/COPD.S172639 PMC6171757

[21] Kaczka DW Dellacá RL. Oscillation mechanics of the respiratory system: applications to lung disease. Crit Rev Biomed Eng. 2011;39:337–59. 22011237 10.1615/critrevbiomedeng.v39.i4.60 PMC3237123

[22] Lipworth BJ Jabbal S. What can we learn about COPD from impulse oscillometry? Respir Med. 2018;139:106–9. 29857993 10.1016/j.rmed.2018.05.004

[23] Zerah F Lorino AM Lorino H Harf A Macquin-Mavier I. Forced oscillation technique vs spirometry to assess bronchodilatation in patients with asthma and COPD. Chest. 1995;108:41–7. 7606989 10.1378/chest.108.1.41

[24] Nikkhah M Amra B Eshaghian A. Comparison of impulse osillometry system and spirometry for diagnosis of obstructive lung disorders. Tanaffos. 2011;10:19–25. 25191346 PMC4153135

[25] Kolsum U Borrill Z Roy K. Impulse oscillometry in COPD: identification of measurements related to airway obstruction, airway conductance and lung volumes. Respir Med. 2009;103:136–43. 18760576 10.1016/j.rmed.2008.07.014

